# Initial evidence regarding the neurobiological basis of psychological symptoms in dementia caregivers

**DOI:** 10.1038/s41398-023-02457-8

**Published:** 2023-05-18

**Authors:** Stephen F. Smagula, Howard J. Aizenstein

**Affiliations:** Department of Psychiatry, School of Medicine, University of Pittsburgh, Pittsburgh, PA USA

**Keywords:** Prognostic markers, Neuroscience

## Abstract

Mood symptoms and disorders are common in dementia caregivers, who can be exposed to a myriad of potential stressors including their care recipient’s neuropsychiatric symptoms. Existing evidence indicates that the effects of potentially stressful exposures on mental health depend on the caregiver’s individual characteristics and responses. Specifically, prior studies indicate that risk factors measured on psychological (e.g., emotion-focused/behaviorally disengaged coping responses) and behavioral (e.g., sleep and activity restriction) levels of analysis may confer the effects of caregiving exposures on mental health. Theoretically, this process from caregiving stressors and other risk factors to mood symptoms is neurobiologically mediated. This article reviews recent studies that used brain imaging to identify neurobiological factors that are related to psychological outcomes in caregivers. Available observational data indicate that psychological outcomes in caregivers are related to differences in the structure/function of regions involved in socio-affective information processing (prefrontal), autobiographical memory (the posterior cingulate), and stress (amygdala). In addition, two small randomized controlled trials using repeated brain imaging showed that Mentalizing Imagery Therapy (a mindfulness program) increased prefrontal network connectivity and reduced mood symptoms. These studies raise the possibility that, in the future, brain imaging may be useful to detect the neurobiological basis of a given caregiver’s mood vulnerability and guide the selection of interventions that are known to modify it. However, there remains a need for evidence on whether brain imaging improves on simpler/inexpensive measurement modalities like self-report for identifying vulnerable caregivers and matching them with efficacious interventions. In addition, to target interventions, more evidence is needed regarding the effects that both risk factors and interventions have on mood neurobiology (e.g., how persistent emotion-focused coping, sleep disruption, and mindfulness affect brain function).

Due to a myriad of factors, being a dementia caregiver (dCG) can be extremely stressful. As such, it is not surprising that an estimated 1 in 5 dCGs have a depressive disorder [1]. Depression symptoms are consequential in dCGs, including by their relationship with poorer quality caregiving [2] and higher risk for chronic diseases [3–7]. But even in these studies which demonstrate high rates of depression and anxiety in dCGs, the evidence is clear: not all dCGs experience significant mood disturbance. Indeed, a recent report from the *National Academies of Sciences, Engineering, and Medicine* titled “Families Caring for an Aging America” [8] noted that the impact of caregiving on mental health is “highly individualized.” What is protecting some, but not all, dCGs from depression?

Several conceptual models have been developed to explain individual variability in the psychological consequences of caregiving [9–13]. Common across these conceptual models is the idea that individual differences, e.g., in circumstances, characteristics, and/or responses, determine whether caregivers will experience mood disturbances. Considerable research in this area has examined individual differences in terms of caregiving exposures, as well as psychological (e.g., coping styles), and behavioral (e.g., insufficient sleep) risk factors. In theory, these effects of stressful caregiving exposures and other risk factors on mood are neurobiologically mediated. But only relatively recently have studies been able to use MRI to examine how individual differences in dCG’s brain structure and function relate to their mental health.

This review describes the findings from initial studies that used brain MRI to examine relationships between dCG’s neurobiology and psychological outcomes. To provide context, we first review the types of stressors that caregivers may face, as well as evidence from studies that have identified psychological and behavioral factors that are associated with worse mood outcomes in caregivers. As a guide, we provide a simplified conceptual model (Fig. 1) that distinguishes potentially stressful exposures, from individual factors/characteristics that may mediate their effects (measurable across levels of analysis), leading to negative outcomes like depression.

**Fig. 1:**
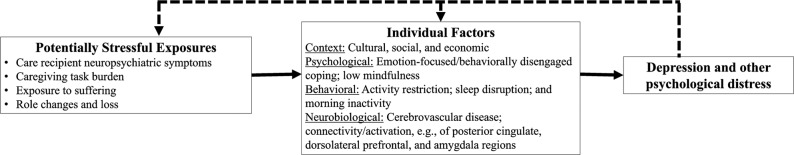
Conceptual model. Simplified conceptual model distinguishing potentially stressful exposures, individual factors/characteristics that may mediate their effects measurable across levels of analysis, leading to negative outcomes like depression.

## Potentially stressful exposures facing dementia caregivers

A major potentially stressful exposure for dCGs is the presence of neuropsychiatric symptoms in the care recipient (left of Fig. 1). Neuropsychiatric symptoms have been consistently linked with worse mental health outcomes among dCGs [14–17]. Common neuropsychiatric symptoms in people with dementia include depression and apathy, as well as behavioral disruption such as irregular sleep patterns. Aggressive or dangerous actions such as wandering can also occur in people with dementia.

Differences in regional neurodegeneration among dementia care recipients have been linked with their neuropsychiatric symptoms and caregiver psychological symptoms [18]. This study found greater patient atrophy in right insula and superior medial frontal areas was related to worse caregiver health. Furthermore, the relationship between lower patient grey matter in these regions and caregiver health was partially statistically mediated by patient neuropsychiatric symptoms. Importantly, the authors noted that patient cognitive function did *not* statistically mediate the association of patient grey matter and caregiver health. Compared with a model including neuropsychiatric symptoms, a model additionally including brain volume data explained more variance in caregiver psychopathology symptoms. Based on these findings, these authors suggested that, in the future, measures of regional grey matter degeneration may provide clinical teams with a warning sign (e.g., that the patient may be more likely to have neuropsychiatric symptoms and/or that the caregiver may be more likely to experience mental distress).

It is important to note that in addition to neuropsychiatric symptoms, several other factors contribute to variability in stress among dCGs. Caregivers may differ in terms of the level of care that they provide, including management of complex medical routines, provision of physical assistance, and if they feel “on call 24/7” to support safety and a broad range of daily functions. Care regimes, once established, may need to be revised as dementia progresses and functional decline occurs. In addition, caregivers may be highly exposed to their care recipient’s physical/emotional suffering. Difficult role and relationship changes may occur. All of these potentially stressful exposures theoretically contribute to feelings of distress/grief about their care recipient’s condition, loss of function, anticipation of further decline, and death.

## Individual factors linked with mental health in dementia caregivers

Evidence suggests that the negative psychiatric effects of caregiving-related exposures depend upon factors that vary across individuals (middle of Fig. 1). Note that the individual differences examined as potential risk factors in research cross several levels of analysis. First, note that differences in contextual factors may enhance or mitigate negative health outcomes among caregivers. As reviewed previously [12], relevant contextual factors include an individual’s race, culture, the availability of social support, and financial resources.

In addition, several studies have examined psychological coping, in particular emotion-focused and/or behaviorally disengaged coping, as a mediator between caregiving exposures and psychological/mood outcomes [19–25]. These studies all included measures of the caregiving context as covariates in statistical models to demonstrate that, above and beyond exogenous factors like caregiving intensity or care recipient behaviors, caregivers with emotion-focused, disengaged, or escape/avoidant coping responses had worse mental health outcomes. Some of these studies explicitly modeled statistical mediation to demonstrate that associations between care recipient behavioral problems and mood outcomes could be statistically accounted for by differences in the degree of emotion-focused coping responses [19–21].

Finally note that several studies have considered disruption to health behaviors, including sleep and activity, as correlates of mood disturbances in dCGs. Caregivers often report being unable to participate in meaningful/rewarding activities due to their caregiving role; this type of activity restriction has been associated with depression among caregivers in meta-analysis [26]. In addition, the majority (50–74%) of dCGs report sleep disturbances [27]. One meta-analysis estimated that, on average, dCGs slept 23–33 minutes a night less than age-matched non-caregiving adults [28]. Our prior study found that several of these behavioral factors correlated cross-sectionally with depression symptoms, including: self-reported activity restriction, self-reported insomnia severity, actigraphy measured sleep fragmentation, and actigraphy measured inactivity in the morning hours from 8–10 AM [29]. In a multivariable model including these sleep and activity characteristics, only morning inactivity predicted depression symptom persistence six-months later. While we are unaware of empirical data linking morning inactivity with the use of emotional-focused coping, we propose these behavioral and psychological factors may be on the same pathway (e.g., “lingering” in bed the morning co-occurring with focus on negative emotions).

## Neurobiological measures related to mental health in dementia caregiving

We are only aware of four studies to date that examined caregiver brain measures in relation to their mental health (Table 1). In 2017, we reported results from a secondary analysis of data collected in the early 1990s [30]. The sample consisted of 237 co-residing spousal caregivers who were not necessarily dementia caregivers. We analyzed whether visually rated features of brain pathology were associated with a greater likelihood that participants reported caregiving strain. Findings showed a relationship between higher white matter grades, reflecting greater cerebrovascular disease, and caregiver strain. Among caregivers with higher white matter grades (indicating cerebrovascular disease), about 80% reported strain (compared with ~50% of caregivers who had lower white matter grades). There was also a statistical interaction between white matter grade and the level of functional assistance provided by the caregivers. Among caregivers with lower white matter grades, caregiving strain was more common for those who provided more functional assistance to their care recipient. However, in caregivers with high white matter grades (indicating possible white matter damage), rates of strain were high regardless of the level of functional assistance that they provided.

**Table 1:** Summary of MRI findings in dementia caregivers.

Observational		
Year	Sample	Findings
2017	237 spousal caregivers	Caregivers were more likely to report strain if they had high white matter grades indicating possible damage to white matter integrity of cerebrovascular origin
2019	17 dementia caregivers	Viewing pictures of care recipients vs. strangers elicited greater dorsal anterior cingulate and posterior cingulate cortex activation. In response to pictures of care recipients, caregivers with higher mindfulness scores had greater engagement of prefrontal, middle temporal, precuneus, and cerebellar regions.
2020	41 dementia caregivers	Caregivers were more likely to have at least mild depression symptoms if they had lower white matter integrity in tracts: connecting dorsolateral prefrontal and subgenual anterior cingulate; and tracts connecting to posterior cingulate cortex.
2021	54 dementia caregivers	A lack of “morningness” was associated with higher resting state connectivity between amygdala and posterior cingulate regions, which statistically mediated the association of low “morningness” with depression symptoms
Interventional		
Year	Sample	Findings
2012	9 dementia caregivers	Changes in brain metabolism differed between caregivers who were randomized to relaxation versus meditation. The meditation group had decreases in inferior frontal cortex and posterior cingulate metabolism, whereas the relaxation group had increased metabolism in these regions.
2021	24 dementia caregivers (16 with imaging)	Mentalizing Imagery Therapy (versus a control group) was associated with improved mental health and increased resting state connectivity of the dorsolateral prefrontal cortex.
2022	46 dementia caregivers (n = 28 with imaging)	Results replicated 2021 study (described in the row above) and further showed that increases in resting state dorsolateral prefrontal network connectivity correlated with decreased depression symptoms and increases in trait mindfulness.

In 2019, Jain et al., reported findings relating brain responses during a functional brain imaging task (fMRI) to mental health measures in 17 family dCGs [31]. Caregivers provided photographs of their care recipients who had dementia. The fMRI task included one factor comparing brain responses to seeing their loved one versus a picture of a stranger (picture condition). Relative to viewing strangers, care recipient pictures elicited greater activation in the dorsal anterior cingulate gyrus and posterior cingulate cortex. These authors suggested that dorsal anterior cingulate activation in response to pictures of their care recipients may be related to a social pain response, and that posterior cingulate activation may be related to the care recipient picture’s being autobiographically relevant. Furthermore, the authors examined how these brain activation responses differed based on grief, mindfulness, and depression symptoms related to differences in brain activation during the picture conditions.

These authors [31] noted that dementia caregivers with higher mindfulness scores had greater brain activation in several regions in the picture condition (seeing care recipient versus stranger pictures). Specifically, dCGs with greater mindfulness score had more engagement of prefrontal, middle temporal, precuneus, and cerebellar regions in the picture condition. The authors note that the engagement of these regions in the dCGs with greater mindfulness scores may reflect sustained attention while processing potentially emotionally negative autobiographically relevant stimuli. Note that in this sample, neither grief nor depression severity was related to statistically significant differences in picture task activation. Mindfulness scores did correlate moderately with lower depression symptom severity and strongly with lower levels of grief.

In 2020, we published results comparing dCGs with and without mild depression symptoms (*n* = 41) in terms of the structural integrity of several white matter tracts [32]. Forty-nine percent of this baseline sample had Patient Health Questionnaire—9 scores of 5 or more which indicates that they had at least mild depression symptoms [33] (which are health relevant due to their potential negative effects [3] and association with the incidence of major depressive disorder [34, 35]). We compared dCGs with and without mild depression symptoms in terms of the fractional anisotropy of white matter tracts (reflecting structural integrity) connecting to 11 distinct regions that were previously implicated in depression studies. The group of dCGs with mild depression symptoms had relatively lower fractional anisotropy in tracts connecting the dorsolateral prefrontal and rostral cingulate cortices; and in tracts connecting to the posterior cingulate cortex. We suggested that lower integrity white matter connecting to frontal and rostral cingulate regions may impair emotional reappraisal; and that lower integrity white matter connecting to the posterior cingulate might compromise autobiographical information processing.

In 2021, we published a resting-state study [36] using an expanded version of the dCG sample described in the paragraph above. Fifty-four dCGs had adequate resting-state fMRI data. We examined the resting-state functional connectivity of regions previously implicated in depression including the dorsolateral prefrontal cortex, amgydala, posterior cingulate, and anterior insula. Based on our finding (reviewed above) that linked morning inactivity with mood in dCGs [29], we examined if resting-state connectivity differed between “morning type” and “non-morning type” caregivers. Results showed that non-“morning type” dCGs had relatively greater resting state connectivity between the amygdala and ventral posterior cingulate. Higher resting-state amygdala-ventral posterior cingulate cortex connectivity also statistically mediated the association between a lack of “morningness” and greater depression symptoms. We interpreted these findings based on prior studies that show amygdala activation related to avoidance and escape related responses [37] and posterior cingulate involvement in self-referential processing including thinking about one’s duties [38]. We thus suggested that higher resting amygdala-PCC connectivity observed in dCGs with morning inactivation may reflect persistent negative emotional self-referential attention, e.g., due to rumination or emotion-focused coping.

## Effects of interventions on dementia caregiver neurobiology

In 2012, Pomykala et al. reported results from a pilot randomized controlled trial examining the effects of meditation training versus relaxation [39]. Dementia caregivers were assigned to do 12 min of meditation (*n* = 4) or relaxation (*n* = 5) each day for eight weeks. Using repeated FDG-PET scans, the authors assessed changes in resting brain metabolism before and after the intervention. The observed differences differed by group assignment. The two largest differences in brain metabolism changes between the two groups were that: (1) inferior frontal cortex metabolism decreased in the meditation group, but increased in the relaxation group; and (2) posterior cingulate metabolism decreased in the meditation group, but increased in the control group. The authors suggested that the changes observed may be due to the development of greater resting state metabolic efficiency in these regions due to meditation.

In 2021 Jain et al. published a pilot study involving 24 family dCGs who had at least moderate depression symptoms [40]. These dCGs were equally randomized to a wait list/control or the active mindfulness training condition called Mentalizing Imagery Therapy (MIT). The active treatment MIT group had greater improvements in mental heath measures than the control group. The MIT group (but not the control group) also had increased resting-state connectivity within a network containing the dorsolateral prefrontal cortex.

Following up on these findings in 2022, Jain et al. reported results from a trial of dCGs randomized to MIT (*n* = 24) or a support group control (*n* = 22). Again, relative to the control, MIT resulted in improved psychological symptoms. In 28 participants with imaging data, the authors examined changes in resting state connectivity of two networks: one with bilateral dorsolateral prefrontal cortex as a major node; and one with subgenual anterior cingulate cortex as a major node. Reproducing findings from their pilot (described above), results showed that MIT (but not the control) was associated with increases in resting connectivity within the dorsolateral prefrontal cortex network. Furthermore, in the sample overall, dorsolateral prefrontal cortex network connectivity increases correlated with improvements in depression symptoms and increases in trait mindfulness.

## Summary and conclusion

This article began by reviewing salient stressors and maladaptive responses which may increase the risk that dCGs experience psychological symptoms. Evidence indicates that neuropsychiatric symptoms in care recipients and emotion-focused/behaviorally disengaged coping responses are associated with caregivers having more psychological symptoms. As summarized here (see Table 1), studies have also now begun characterizing the neurobiological correlates of psychological symptoms in dCGs. These brain imaging studies have suggested that psychological outcomes in dCGs may be related to differences in the structure/function of regions subserving socio-affective information processing (prefrontal [31, 32]), autobiographical memory (the posterior cingulate [31, 32]), and stress (amygdala [36]).

Causal pathways linking caregiving stress, psychological/behavioral risk factors, and mood neurobiology are plausible. For example, it is plausible that emotion-focused coping responses lead to sleep disruption, e.g., by increasing negative emotional arousal after care-related awakenings. In turn, morning inactivity/fragmentation of rapid eye movement sleep (which occurs predominately in later sleep cycles/early morning hours [41]) may promote ongoing amygdala activation and distress [42]. Other links between psychological/behavioral factors and neurobiological mediators are also plausible. For example, initial studies in dCGs show that mindfulness practices, which deploy focused attention/increase non-judgmental accepting awareness, can affect prefrontal network connectivity strength [40, 43], and this could translate into greater use of problem-focused coping and mood resilience. While these pathways linking traditional psychological/behavioral factors with mood neurobiology are plausible, future studies are needed to evaluate these hypotheses about how risk factors measured across multiple levels of analysis inter-relate and uniquely contribute to shape mood outcomes and treatment responses.

Achieving a transdisciplinary understanding of why many, but not all, dCGs develop significant mood disturbances could facilitate more targeted screening and deficit-based interventions. Indeed, in 2012 Pomykala et al [39]. suggested that “more research in this area can enrich clinical practice and help individualize and tailor behavioral interventions to individual brain metabolic profiles.” Now a decade later, research in this area remains promising but still new. The brain imaging studies of dementia caregivers reviewed here have been few in number, and based on relatively small sample sizes which may not be representative of all dementia caregivers. These studies have yet to demonstrate that the relatively high cost and burden of brain imaging will be warranted in practice, i.e., whether brain imaging data significantly improves on questionnaires in terms of detecting mood vulnerability or selecting of interventions that modify it. Future studies are needed to determine which combination(s) of psychological, behavioral, and neurobiological data optimally predict which caregivers are susceptible to mood disturbances and who will respond to particular treatments. In addition, to help guide the development of neuroscience-based precision intervention approaches, future studies are needed to establish how risk factors affect mood neurobiology and which interventions work on these processes.
